# PLACE OF ORIGIN AND USE OF FORMAL HEALTH SERVICES

**DOI:** 10.1093/geroni/igae098.1673

**Published:** 2024-12-31

**Authors:** Allen Glicksman, Steven Albert, Adam Davey, Ari Friedman, Philip Lai, Misha Rodriguez, Robert Schrauf, Michael Liebman

**Affiliations:** NewCourtland, Philadelphia, Pennsylvania, United States; University of Pittsburgh, Pittsburgh, Pennsylvania, United States; University of Delaware, Newark, Delaware, United States; University of Pennsylvania, Philadelphia, Pennsylvania, United States; Philadelphia Senior Center, Philadelphia, Pennsylvania, United States; NewCourtland, Philadelphia, Pennsylvania, United States; The Pennsylvania State University, University Park, Pennsylvania, United States; IPQ Analytics. LLC, Kennett Square, Pennsylvania, United States

## Abstract

The point of contact between older immigrants and the formal health system is an area of concern for providers, policy makers and researchers. The challenges that often appear in any interaction between older adults and formal providers are exacerbated when the older person is an immigrant because of language and cultural differences, challenges in a family where migration has taken place, and lack of familiarity with the local system for providing care. Attempts to understand these interactions require attention to two sources of diversity – on the one hand the place of origin of the older migrant and all the social, cultural and policy differences in the places of origin on the one hand and the diversity of types of encounters (ambulatory, emergency department, in-patient hospital) with the formal system and the related issues of insurance status on the other. Our study, using data on encounters between persons speaking Chinese, Spanish or Russian to examine use of the three types of encounters to examine patterns of service use for persons with similar health insurance and primary diagnoses. We will focus on the Chinese speakers and their distinctive pattern of use of formal health service compared to the other groups (Including white and Black English speakers). This is especially true in the lower rate of using emergency departments compared to the other groups. We will also address the challenges to studying immigrant experience in health settings when only language of interaction and not place of birth are collected by health providers.

